# Rationale and design to evaluate the PRIME Parkinson care model: a prospective observational evaluation of proactive, integrated and patient-centred Parkinson care in The Netherlands (PRIME-NL)

**DOI:** 10.1186/s12883-021-02308-3

**Published:** 2021-07-23

**Authors:** Jan H. L. Ypinga, Angelika D. Van Halteren, Emily J. Henderson, Bastiaan R. Bloem, Agnes J. Smink, Emma Tenison, Marten Munneke, Yoav Ben-Shlomo, Sirwan K. L. Darweesh

**Affiliations:** 1 Radboud University Medical Centre, Donders Institute for Brain, Cognition and Behaviour, Department of Neurology, P.O. Box 9101, 6500 HB Nijmegen, The Netherlands; 2 Department of Population Health Sciences, Bristol Medical School, University of Bristol, Bristol, BS8 1NU UK; 3 Older People’s Unit, Royal United Hospitals Bath NHS Foundation Trust, Combe Park, Bath, UK

**Keywords:** Parkinsonism, Parkinson’s, Disease, Integrated Care Model, Care Management

## Abstract

**Background:**

Culminating evidence shows that current care does not optimally meet the needs of persons with parkinsonism, their carers and healthcare professionals. Recently, a new model of care was developed to address the limitations of usual care: Proactive and Integrated Management and Empowerment in Parkinson’s Disease (PRIME Parkinson). From 2021 onwards, PRIME Parkinson care will replace usual care in a well-defined region in The Netherlands. The utility of PRIME Parkinson care will be evaluated on a single primary endpoint (parkinsonism-related complications), which reflects the health of people with parkinsonism. Furthermore, several secondary endpoints will be measured for four dimensions: health, patient and carer experience, healthcare professional experience, and cost of healthcare. The reference will be usual care, which will be continued in other regions in The Netherlands.

**Methods:**

This is a prospective observational study which will run from January 1, 2020 until December 31, 2023. Before the new model of care will replace the usual care in the PRIME Parkinson care region all baseline assessments will take place. Outcomes will be informed by two data sources. We will use healthcare claims-based data to evaluate the primary endpoint, and costs of healthcare, in all persons with parkinsonism receiving PRIME Parkinson care (estimated number: 2,000) and all persons with parkinsonism receiving usual care in the other parts of The Netherlands (estimated number: 48,000). We will also evaluate secondary endpoints by performing annual questionnaire-based assessments. These assessments will be administered to a subsample across both regions (estimated numbers: 1,200 persons with parkinsonism, 600 carers and 250 healthcare professionals).

**Discussion:**

This prospective cohort study will evaluate the utility of a novel integrated model of care for persons with parkinsonism in The Netherlands. We anticipate that the results of this study will also provide insight for the delivery of care to persons with parkinsonism in other regions and may inform the design of a similar model for other chronic health conditions.

## Background

Parkinsonism, which aside from its most common subtype Parkinson’s Disease (PD) includes various forms of atypical parkinsonism, is a rapidly growing source of disability globally [1, 2]. Current care is not designed optimally to meet the specific needs of persons with parkinsonism [3, 4]. To understand the specific challenges in current care for parkinsonism, several qualitative and quantitative pilot investigations have been conducted over the last few years, revealing specific unmet needs of persons with parkinsonism, their carers and healthcare professionals involved in their care [5–11]. In particular, usual care for persons with parkinsonism is constrained by a lack of multidisciplinary collaboration and poor continuity, delayed detection and reactive management of symptoms, and difficulties in accessing healthcare professionals with appropriate expertise [12]. Furthermore, there is a lack of empowerment and involvement for persons with parkinsonism and carers, care is not managed close to home, and care typically follows a ‘one size fits all’ approach [12]. These limitations not only negatively influence quality of life and associated other measures of health of people with parkinsonism (reflecting the ‘health’ dimension), but also have a negative economic impact (‘cost of healthcare’) as well as burdening persons with parkinsonism and carers (‘patient and carer experience’) and impairing professional fulfilment of healthcare professionals (‘healthcare professional experience’) [9, 13, 14]. In combination, these four dimensions reflect a previously described framework to evaluate the quality of care (‘Quadruple Aim’) [15, 16].

Recently, the recognition of these challenges has been used to develop an integrated care model that focuses on regional collaboration: Proactive and Integrated Management and Empowerment in Parkinson’s Disease (PRIME Parkinson) care [17]. The concept of PRIME Parkinson care aims to address the unmet needs of persons with parkinsonism and carers, while also accommodating preferences of healthcare professionals involved in their care as much as possible and keeping the costs neutral. The components of PRIME Parkinson care are summarized in Table 1. PRIME Parkinson care will be administered to persons with parkinsonism irrespective of whether their underlying disease is PD or atypical parkinsonism, because of the large overlap in problems that persons with these diseases face, and also because there is always a certain degree of diagnostic uncertainty when it comes to the clinical distinction between PD and atypical parkinsonism [18, 19].

**Table 1:** Core elements of the PRIME Parkinson care intervention

Usual care	PRIME Parkinson care
Lack of multidisciplinary collaboration and continuity of care	Deliver integrated care and continuity of care
Issues detected late and managed reactively	Manage issues early and proactively
Difficult to access healthcare professionals with appropriate expertise in a timely fashion	Facilitate access to specialised healthcare professionals
Lack of empowerment and involvement for persons with parkinsonism and carers	Educate and empower persons with parkinsonism and carers
Care not managed close to home	Organise care close to home
'One size fits all' treatment and focus mainly on motor symptoms	Deliver personalised care and "precision" medicine

Note that we have previously published a full description of the PRIME Parkinson care intervention [17]

The purpose of this paper is to describe how the research evaluation of PRIME Parkinson care in the Netherlands (PRIME-NL) has been designed. There is a parallel but independent research evaluation in the United Kingdom which will reported elsewhere and follows the same guiding principles but with a different methodology. PRIME-NL will be implemented for persons with parkinsonism in the PRIME Parkinson care region from 2021 onwards, replacing usual care for all persons with parkinsonism in the region; it will not be implemented as a research intervention [12]. We will prospectively evaluate the utility of PRIME Parkinson care, compared to usual care in other regions of The Netherlands. The evaluation will span each dimension of the Quadruple Aim framework: health, patient experience, healthcare professional experience, and cost of healthcare [15, 16]. As the model also targets the quality of care for carers of persons with parkinsonism, we will additionally evaluate the model’s utility on a fifth dimension: carer experience.

## Methods

### Overview of study design and data sources

This is a prospective observational study, which will run from January 1, 2020 through December 31, 2023. The study comprises two data sources: healthcare claims-based data and questionnaire-based data. We will use healthcare claims-based data to compare all persons with parkinsonism in the PRIME Parkinson care region (Index sample) with all persons with parkinsonism across other regions in The Netherlands (Control sample). The choice to include four community hospitals in the PRIME Parkinson care region was determined by the available funding. The choice for these specific hospitals was based on their geographical proximity (i.e., a 25 km radius) to the Radboud University Medical Centre, as well as a prior history of collaboration in clinical and research settings [20, 21].

**Fig. 1:**
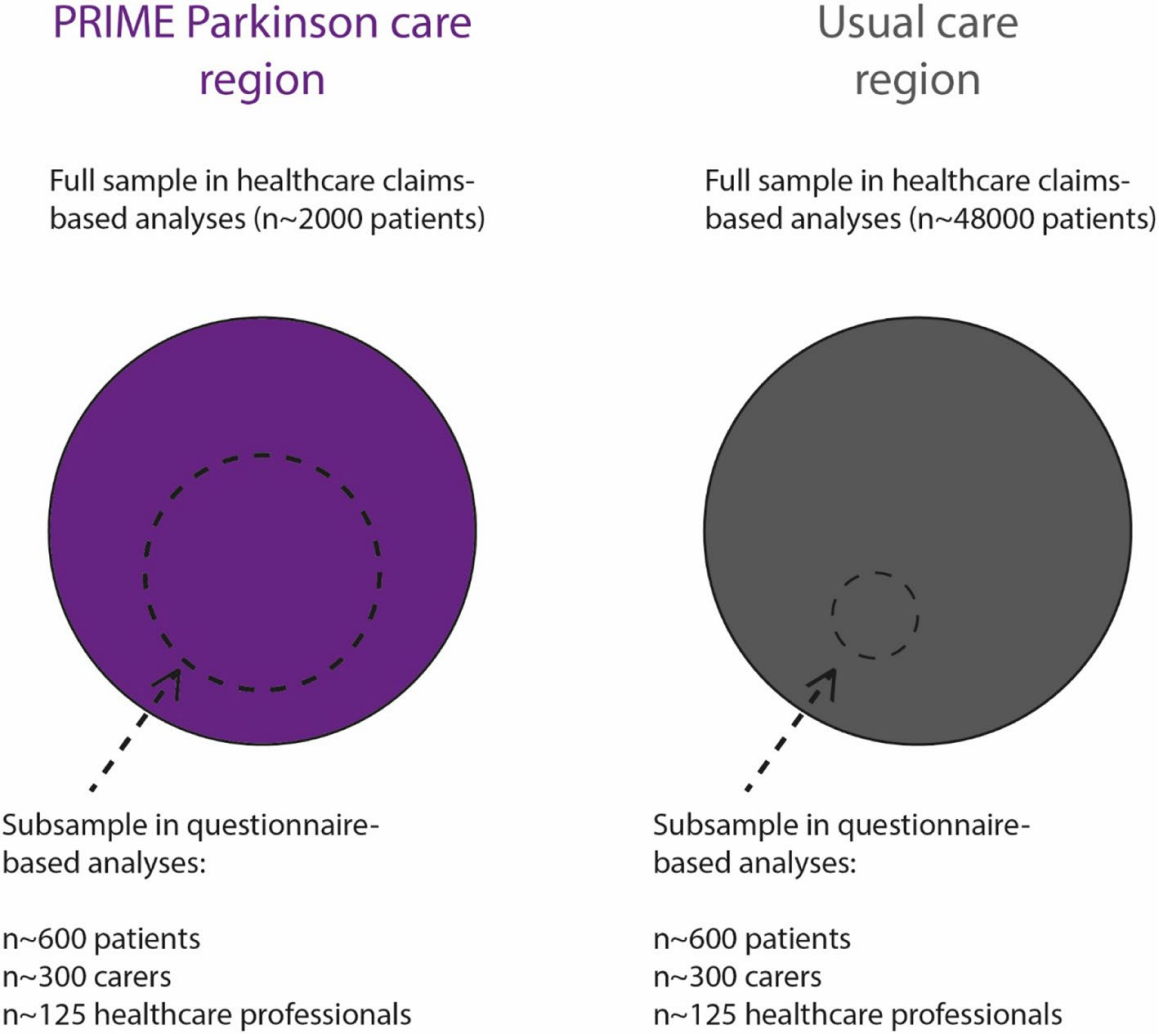
Overview of study population

Furthermore, we will perform annual questionnaire-based assessments in a subsample which will be approximately equally divided over the PRIME Parkinson care and usual care regions (estimated numbers: 1,200 persons with parkinsonism, 600 caregivers and 250 healthcare providers). Since PRIME Parkinson care will be implemented in the Index region from January 1, 2021 onwards, we consider the first year (January 1, 2020 through December 31, 2020) as the baseline observational period and the following three years (January 1, 2021 through December 31, 2023) as the implementation period. An overview of the patient study population is provided in Fig. 1.

We note that both data sources serve complementary purposes: we will use healthcare claims-based data to evaluate the primary endpoint of the health dimension, which is a composite measure of parkinsonism-related complications, as well as other endpoints of health and cost of healthcare. We will use questionnaire-based data to evaluate other endpoints of health, patient and carer experience and healthcare professional experience (Table 2).

**Table 2:** Data sources, categorised by dimension

Dimension	Data source(s)
Health^a^ *(contains the primary endpoint: parkinsonism-related complications)*	Healthcare claims & Questionnaires
Cost of health care	Healthcare claims
Patient and carer experience	Questionnaires
Healthcare professional experience	Questionnaires

^a^Of note, this dimension reflects health of people with parkinsonism

#### Healthcare claims-based data

Healthcare claims-based data will be provided by Vektis, which is the Dutch health care information centre and has healthcare expenditure data on all insured individuals in The Netherlands, which embodies > 99.8% of the Dutch population (± 17.2 million people) [22, 23].

#### Questionnaire-based data

Participants will self-administer questionnaires electronically, with the exception of the telephone Montreal Cognitive Assessment (MoCA) which will be conducted over the phone for all participants. Participants who are not able to complete electronic questionnaires will be provided with two alternatives: paper-based self-administration or telephone-based interview administration. We will conduct annual assessments between 2020 and 2023, i.e. one baseline and three follow-up assessments at 12, 24 and 36 months after enrolment.

#### Eligibility criteria and definition of regions

**Fig. 2:**
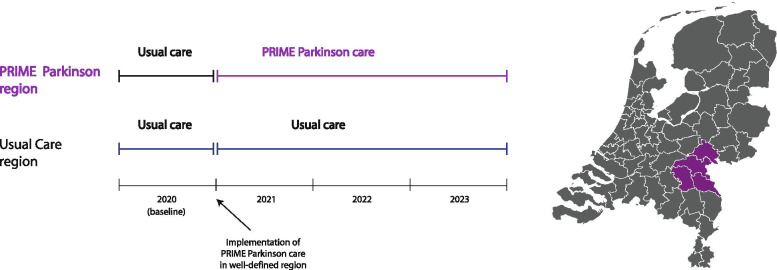
Overview of PRIME Parkinson care and usual care regions and study timeline

Persons with a clinical diagnosis of parkinsonism, except those in whom the cause is medication use (which is potentially reversible), who receive their treatment in a community hospital will be eligible for the study. In this context we refer to non-university medical centres as a community hospital. The PRIME Parkinson care region consists of four community hospitals: Bernhoven Ziekenhuis, Canisius Wilhelmina Ziekenhuis, Maasziekenhuis Pantein, Rijnstate Ziekenhuis. In Fig. 2 the PRIME Parkinson care region is presented visually. Persons who receive parkinsonism treatment in the PRIME Parkinson care region will be compared to persons in a community hospital outside the above-defined region who receive usual care for parkinsonism. There are 60 community hospitals in the usual care region. In both groups, the targeted population is representative of the broad spectrum of persons with parkinsonism who are treated in a community hospital.

Detailed inclusion criteria for persons with parkinsonism, carers and healthcare professionals are presented in Table 3. Carers are eligible to participate even if the person with parkinsonism whom they care for does not participate in this study. For those carers, we have added questions on the characteristics of the person with parkinsonism for whom they provide care.

**Table 3:** Inclusion criteria

*Research population*	Inclusion criteria
Persons with parkinsonism	Have a clinical diagnosis of parkinsonism, except drug-induced parkinsonism; have visited the Neurology outpatient clinic of a community hospital centre at least once during the last year; willing and able to provide informed consent^a^
*Carers*	Be designated as the primary carer by a patient who is eligible to participate; willing and able to provide informed consent^a^
*Healthcare professionals*^b^	Work as a certified nurse, physical therapist, occupational therapist, speech-language therapist or neurologist; have seen at least 5 persons with parkinsonism in the previous year; willing and able to provide informed consent^a^

^a^For inclusion in questionnaire-based assessments only. Carers are eligible for participation even if the person with parkinsonism who they care for does not participate in this study. For those carers, we have added additional questions on characteristics of the person with parkinsonism for whom they provide care. ^b^While it is not feasible to assess healthcare professionals from each field of expertise involved, we intend to longitudinally assess Healthcare Professional Experience in a sample that reflects that diverse composition, comprising nurses, physical therapists, speech-language therapists, occupational therapists and neurologists

This is a closed cohort study, meaning there will be no further inclusion after the baseline assessment round.

#### Recruitment process in the questionnaire-based study

The recruitment of persons with parkinsonism comprises three phases: actively informing potential participants (phase 1), providing additional information to those who are interested (phase 2), and obtaining informed consent (phase 3).

Phase 1 entails publicising the study through various channels. First, invitation letters will be sent to members of ParkinsonNEXT, a web-based platform for persons with parkinsonism and their carers who have expressed an interest in participating in research. Second, the Parkinson Vereniging (Dutch Parkinson patient association) will send newsletters and share posts on their website. Third, a brochure with a reply card has been designed that will be shared with potential participants at different events organized for persons with parkinsonism or their carers, including -among others- ParkinsonNEXT innovation and research events and the national ParkinsonNet congress. One additional recruitment strategy will be applied in the PRIME Parkinson care region only, to increase the likelihood that the target sample size in that region will be reached: hospital-based neurologists in the PRIME Parkinson care region will send information letters to all persons with parkinsonism affiliated to their hospital.

In phase 2 persons with parkinsonism can indicate their interest to receive more information about the study on the study website (https://www.parkinsonnext.nl/prime/) or by contacting the assessment team via telephone, email or sending a reply card by post. Persons with parkinsonism can only participate if they have provided informed consent. Persons with parkinsonism who express an interest by calling or responding via the website will be provided with more information about the study by a call from a member of the research team. In phase 3, persons with parkinsonism who indicate continued interest in participating in the study during the call will receive an information letter and consent form by e-mail or post. Persons with parkinsonism can then sign the informed consent form. Given the focus of our recruitment strategies, we anticipate that the majority of carers will be informal carers of a person with parkinsonism, however, we cannot rule out that some carers had formal training or are professional carers. The PRIME Parkinson Care model will replace usual care in the designated region. This means that, in theory, all persons with parkinsonism will receive the PRIME Parkinson Care model. However, we consider it unlikely that uptake of each component of the model will be complete. There has been preliminary work and ongoing comparable studies which suggest how the uptake/recruitment of the evaluation of the new model of care itself might work out. In pilot investigations of individual components of the model the dropout rate was 5% after 8 months [20]. Based on preliminary work and comparable ongoing studies, we expect that it is achievable to generate the necessary participation and to be able to obtain commitment from participants for the duration of the study [20, 21].

The recruitment process for carers will be almost identical to the recruitment process for persons with parkinsonism, with one exception: we will also inform carers about the study through participating persons with parkinsonism. After informed consent has been provided, persons with parkinsonism are contacted by telephone and asked if they have a carer and whether this person is potentially also interested in participating in this study.

Healthcare professionals will be asked to participate through an invitation mail that is sent out via ParkinsonNet, a community-based professional network of healthcare providers treating persons with parkinsonism [24, 25]. Also, healthcare professionals are asked to sign up via the website or to call the assessment team if they are interested in participating. When a healthcare professional indicates continued interest in participating in the study during the call, an information letter and consent form for healthcare professionals will be sent out by mail and the healthcare professional can then sign the informed consent form.

### Outcome measures

#### Overview

We will compare the utility of PRIME Parkinson care to usual care on five dimensions, each of which covers a single primary endpoint and several secondary endpoints. The single overall primary endpoint of this study is an endpoint that reflects the health dimension: parkinsonism-related complications. Since we anticipate that stand-alone analyses for other dimensions will be conducted, we have also defined a primary endpoint for each dimension, which is presented in the next paragraphs. An overview of all outcome measures and covariates is provided in Table 4.

**Table 4:** Overview of outcome measures in questionnaire-based assessments

*Persons with parkinsonism*		
**Dimension**	**Outcome**	**Instrument**
Health	Quality of life	Parkinson’s Disease Questionnaire-39 (PDQ-39) [26]
	Depressive symptoms	Beck Depression Inventory II [27]
	Anxiety	State Trait Anxiety Inventory for Adults [28]
	Autonomic symptoms	Scales for Outcomes in Parkinson’s Disease—Autonomic Dysfunction (SCOPA-AUT)[29]
	Cardinal motor features	Numeric rating scale for bradykinesia, tremor, rigidity, postural imbalance and Movement Disorders Society Unified Parkinson Disease Rating Scale (MDS-UPDRS) part II on motor functions in daily life [30]
	Freezing	New Freezing of Gait Questionnaire [31]
	Acceptance of Illness	Acceptance of Illness Scale [32]
	Coping strategy	Ways of Coping Questionnaire [33]
	Activities of Daily Living	Self-assessment Parkinson’s Disease Disability score [34]
	Cognitive performance	Telephone Montreal Cognitive Assessment (MoCA) [35]
Patient experience	Integrated Care	Patient Assessment of Chronic Illness Care + (PACIC +) [36]
	Continuity of care	Nijmegen Continuity of care questionnaire [37]
	Self-management	Patient Activation Measurement (PAM) [38]
*Carers*		
Carer experience	Carer burden	Zarit Carer Burden Inventory [39]
	Self-management	Patient-activation measure – Caregivers (PAM-CG) [40]
	Quality of life of Carer	Parkinson’s Disease Questionnaire-Carers [41]
	Coping strategy of Carer	Brief COPE (Coping Orientation to Problems Experienced) [42]
	Social support of Carer	Multidimensional Scale of Perceived social support [43]
*Healthcare Professionals*		
Healthcare professional experience	Healthcare Professional wellbeing	Professional Fulfilment Index [44]
	Integrated Care	Assessment of chronic illness care (ACIC) [45]
	Shared decision making	The 9-item Shared Decision Making Questionnaire (SDM-Q-9) [46]

#### Primary dimension: health

The primary endpoint of the health dimension is the number of parkinsonism-related complications. A parkinsonism-related complication is defined as one of the following events: sustaining a fracture or other orthopaedic injury, urinary tract infection, pneumonia, neuro-psychiatric disorders (delirium, psychosis, hallucinations, mood disorders and anxiety). To determine the occurrence of these events we will use the following proxies in the nationwide healthcare claims-based database from Vektis: having a new (unscheduled) hospital visit or crisis (non-elective) admission, a new drug prescription (for example ATC N06AA codes in the case of a depression) or another type of new care intervention related to a parkinsonism-related complication [47, 48]. Previous studies show a very high specificity but lower sensitivity for the use of Anatomical Therapeutic Chemical (ATC) codes to identity complications, for example a sensitivity of 44–54% and specificity of 89–93% for depression [49]. To raise the sensitivity, we combine Diagnosis-related group (DRG) codes and ATC codes from medical claims data. Several studies show the application of usage of medical claims data in Parkinson's disease research and use DRG and ATC drug prescription claims information as proxies for complications or disease severity [50–52]. For example, the use of ATC N05A and N06D as an indicator for respectively antipsychotics and anti-dementia drugs (which covers both anticholinesterases and other anti-dementia drugs) [52].We have previously used a similar composite endpoint in a healthcare claims-based data study on specialized physiotherapy [53], although we note that the composite endpoint in that study only comprised mobility-related complications and pneumonia. Given the wide scope of targeted preventable complications in PRIME Parkinson care, we have now broadened the definition of that composite endpoint. In secondary analyses on the health dimension which are based on questionnaire-based data, the primary endpoint will be quality of life measured using Parkinson’s Disease Questionnaire-39 (PDQ-39).

#### Secondary dimensions

The primary endpoint of the cost of healthcare dimension is total cost of care, which entails community care, hospital care and pharmaceutical expenditure. Secondary endpoints within this dimension include the distribution of costs over community or hospital care and, separately, costs of parkinsonism-related complications. This approach is similar to our approach in a previously published study [53]. For patient experience the primary endpoint will be Patient Assessment of Chronic Illness Care + (PACIC +)[36]. To evaluate carer experience, the primary endpoint will be Zarit Carer Burden Inventory [39]. The Professional Fulfilment Index [44] will be the primary endpoint for healthcare professional experience. To measure the fidelity and implementation of the elements of the new model of care, which replaces the usual care, the Nijmegen Continuity of care questionnaire will be used as measure for the continuity of care, and the PACIC + which looks at integrated care, and which is a part of Personalized care [36, 37]. Next to the questionnaire data, several process indicators will be used to assess the fidelity of the new model of care: more in-depth data on the usage of the regional PRIME Parkinson helpdesk for persons with Parkinsonism and carers, usage of information databank by professionals, carers and persons with Parkinson and the usage of the Parkinson nurse.

### Covariates

#### Health, cost of healthcare and patient experience

The following potential covariates will be used in analyses on both healthcare claims-based and questionnaire-based data: age, sex, socioeconomic status (based on ecological proxy: postal codes), urbanicity, other comorbidities and proxies for parkinsonism-specific health status. The proxies for parkinsonism-specific health status are operationalized based on years since diagnosis of parkinsonism, parkinsonism specific drugs used ATC code N04 [47], parkinsonism-related complications in the year before enrolment, number of outpatient neurology visits in the year before enrolment, characteristics of the hospital where the majority of the patient’s care is delivered, and number of allied health professional consultations in the year before enrolment [53–59]. For analyses on cost of healthcare, we will additionally adjust for total cost of care in the years prior to the start of this study.

For analyses on questionnaire-based data, we will additionally adjust for: ethnicity, marital status, work status, living situation, urbanicity, age at parkinsonism diagnosis, height and weight, weekly physical activity, smoking, alcohol, caffeine, cognitive performance using a telephone MoCA, complications in the last year, whether a care manager or carer is involved in current care, other comorbidities, and both current and expected quality of care in the participant’s region. Due to the Coronavirus Disease 2019 (COVID-19) crisis in the Netherlands we will also examine the experienced impact of the COVID-19 crisis using a standardized questionnaire about the discomfort that people have experienced due to the COVID-19 pandemic. This 8-item questionnaire describes different situations that may have applied due to COVID-19 related stress. The questionnaire is scored on a 6-point Likert-scale ranging from 0 ('this situation did not occur') to 5 ('very troublesome').

#### Carer experience

The following potential covariates will be used: age, sex, education, marital status, work status, living situation and the carer’s relationship to the patient, as well as patient characteristics.

#### Healthcare professional experience

The following potential covariates will be used: age and sex of the healthcare professional, type of profession, work environment (hospital vs primary care setting), years of experience, number of persons with parkinsonism seen per year by the healthcare professional.

### Statistical analysis

#### Overview

In the next paragraph, we present a global outline and sample size calculation of the primary overall analysis of this study. We intend to separately publish a detailed statistical analysis plan, which will cover -among other items- how the simultaneous occurrence of multiple admissions will be handled, how complications will be weighed, and how the influence of the COVID-19 pandemic will be handled. The detailed statistical analysis plan will also contain our approach and sample size calculations for secondary endpoint analyses, including questionnaire-based analyses.

#### Primary endpoint analysis

We will use a negative binomial regression model in which the difference-in-difference, operationalized as an interaction term between time (pre- or post-implementation of PRIME Parkinson care in selected region) and model of care (PRIME Parkinson care or usual care), is the independent variable of interest and the number of PD-related complications (a count variable) is the dependent variable. All covariates described in the previous section will be entered as fixed effects; additionally, random intercepts and random slopes over time will be included. To handle missing data on potential covariates, we will perform multiple imputation based on age, sex and other potential covariates.

#### Sample size calculation

We estimate that approximately 5% of persons with parkinsonism who receive care at a community hospital in The Netherlands reside in the PRIME Parkinson care region [60]. We estimate that there was no difference between regions in parkinsonism-related complications prior to implementation of PRIME Parkinson care in the selected region, and that -after implementation- PRIME Parkinson care will be associated with a 25% reduction in parkinsonism-related complications compared to usual care. This effect would be slightly higher than the effect of specialized physiotherapy compared to generic physiotherapy in a previous study [53]. In both regions, we expect a similar mortality rate as in the intervention arm of the same previous study (28.9 deaths/1000 person-year)[53] and an emigration rate similar to recent estimates from the Statistics Netherlands (CBS) (9 emigrants/1000 person-year)[61, 62]. Since Vektis has healthcare expenditure-based data on all insured individuals in The Netherlands, we expect attrition due to other causes to be minimal. After correction for expected attrition, we would require a total sample size of 29,990 to have 80% power at alpha 5%. This indicates that the estimated number of persons with parkinsonism in both regions (n ~ 50,000) are more than sufficient to detect a 25% difference in parkinsonism-related complications between regions. Of note, since we have defined a single endpoint for the overall primary analysis of this study, we will not adapt statistical threshold for multiple testing.

## Discussion

In this prospective clinical cohort study we will determine the utility of PRIME Parkinson care in the Netherlands, which is a novel integrated care approach for persons with parkinsonism, their carers and the healthcare professionals who are involved in their care. We will also include an economic evaluation. There are several methodological issues related to the study design that need to be considered. First, given the essential role of regional collaboration in PRIME Parkinson care in the Netherlands, we did not consider it feasible to randomize the type of care to clusters at a hospital level. Therefore, PRIME Parkinson care was not introduced as a research intervention in the index region but rather as a replacement of usual care, and the nature of the evaluation of the utility of the model is observational. Any observed differences on outcomes may therefore reflect confounding by other regional factors be they operating at an individual or structural level. We will attempt to adjust for these factors in our multivariable models. The controlled before and after comparison will also adjust for unmeasured confounding unless they vary over time and by area. In other settings where regional collaboration is already well-developed as part of usual care, a clustered randomized controlled trial may be feasible. Second, we have chosen to include only persons with parkinsonism from non-academic centres in the evaluation of the utility of PRIME Parkinson care. The reason for this choice is that usual care for persons with parkinsonism in community hospitals in the PRIME Parkinson care region is likely reflective of usual care in community hospitals across The Netherlands. By contrast, usual care for persons with parkinsonism at Radboud University Medical Centre, which is a tertiary movement disorder centre, is less reflective of usual care for persons with parkinsonism at other academic centres, with the possible exception of other tertiary movement disorder centres. Third, recruitment strategies are identical across the PRIME Parkinson care and usual care regions, with one exception: treating neurologists in the PRIME Parkinson care region will inform persons with parkinsonism of the possibility of participating in a questionnaire-based evaluation of regional differences in the quality of care. This additional recruitment activity could introduce selection bias if they disproportionally attracted participants prone to an accelerated -or, conversely, decelerated- decline in health. To quantify possible selection bias that may arise because of this difference in recruitment, we will assess differences between regions in the baseline characteristics of participants. Fourth, shortly before the planned initiation of baseline assessments for persons with parkinsonism, carers and healthcare professionals in the spring of 2020, the COVID-19 crisis erupted in The Netherlands, with profound regional differences in the incidence of COVID-19 [63]. We employed several complementary strategies to carefully measure how the COVID-19 crisis may have influenced the evaluation of the model, and adjust for that influence as much as possible. In the questionnaire-based study, we added a questionnaire on the impact of the COVID-19 crisis experienced by persons with parkinsonism, carers and healthcare providers. This questionnaire will allow us to quantify potential confounding or information bias that may arise if there are regional differences in the impact of the COVID-19 crisis. Furthermore, we added a questionnaire to measure whether uptake of some elements of the model was accelerated by the COVID-19 crisis, such as the use of telemedicine tools that allow for a shift of care closer to home. Such an accelerated uptake would be in line with the international experience, suggesting that telemedicine tools such as videoconferencing have been adopted more readily than in the years before [64]. Despite these precautionary measures that we have taken, it remains possible that the COVID-19 crisis may have had residual effects on the 2020 baseline evaluation that are not directly measurable through questionnaires. To further mitigate the possible effects of this confounding influence, we intend to perform a difference-in-difference analysis of outcomes in the PRIME Parkinson care and usual care regions over time, instead of relying on a single baseline measurement, in both the questionnaire-based and insurance claims-based study. For the latter, we will also include data from 2019.

We also consider several issues regarding the external validity of this study. First, since the PRIME Parkinson care model will be administered to persons with PD as well as to persons with atypical parkinsonism, this evaluation will also include both diagnostic categories. As a consequence, we expect results of this study to be largely generalisable to persons with parkinsonism irrespective of their underlying diagnosis. Second, we have chosen to include persons with parkinsonism (and carers) at all stages of parkinsonism, to allow for a sample that is representative of the population of persons with parkinsonism in a community hospital. An inherent consequence of this design is that specific subgroups of persons with parkinsonism who are less prone to frequent outpatient visits, e.g. people who do not have capacity to consent to the study or who have developed cognitive problems or persons living in a nursing home, may be relatively underrepresented across both study arms. The results arising from the PRIME Parkinson care model in the Netherlands will be compared to parallel – but independently acquired – results from the United Kingdom (Bath area). Assuming that the findings across both studies will be consistent, this will provide greater certainty that the underlying model behind PRIME Parkinson has validity and is generalisable to different high-income populations, despite differences in health care systems as well as cultural differences across both regions. The primary analysis of the evaluation of the model in each country will be separate, as some elements in the implementation of the model may differ between The Netherlands and the United Kingdom because of different needs in the current respective healthcare systems. Still, in secondary analyses, we intend to learn from the utility of the model in these heterogeneous settings, in order to enhance the external validity of results.

Further details about PRIME Parkinson care in the United Kingdom (PRIME-UK) will be forthcoming and we publish a prespecified analysis plan combining the Dutch and United Kingdom outcomes where appropriate, which will also yield increased statistical power for the assessment of differences between specific subgroups.

We believe that empirical insight on the potential utility of PRIME Parkinson care may be generalisable to other disorders, as parkinsonism can be regarded as a “model condition” for many other chronic neurological disorders, given its wide-ranging clinical phenotype, involvement of multiple professional disciplines in care, multimodal management (e.g., pharmacotherapy, neurosurgical procedures), the long disease duration, and relatively high prevalence [12]. Furthermore, we hypothesize that due to the proactive and collaborative nature of PRIME Parkinson care, persons will be empowered to function better with parkinsonism, leading to improvements in health and patient experience of care -as well as beneficially affecting carer experience and healthcare professional wellbeing- while maintaining cost neutrality. Should this hypothesis be confirmed, then a financial model for this novel care package will have to be developed to demonstrate sustainability and facilitate possible dissemination and implementation to other regions and other high income countries.

## Data Availability

Not applicable, no empirical data are presented in this manuscript.

## Abbreviations

ATC: Anatomical Therapeutic Chemical
CBS: Statistics Netherlands
COVID-19: Coronavirus Disease 2019 caused by the SARS-CoV-2 (2019-nCoV)
DRG: Diagnosis-related group
MoCA: Montreal Cognitive Assessment
PACIC +: Patient Assessment of Chronic Illness Care +
PD: Parkinson’s Disease
PDQ-39: Parkinson’s Disease Questionnaire-39
PRIME Parkinson: Proactive and Integrated Management and Empowerment in Parkinson’s Disease
PRIME-NL: PRIME Parkinson care in the Netherlands
PRIME-UK: PRIME Parkinson care in the United Kingdom
